# Wastewater-based and case-based surveillance data for monitoring COVID-19 in Germany, 2022–2024

**DOI:** 10.1016/j.dib.2026.112969

**Published:** 2026-06-18

**Authors:** Peter Pütz, Susan Abunijela, Udo Buchholz, Timo Greiner, Ann-Sophie Lehfeld, Alexander Schattschneider, Jakob Schumacher

**Affiliations:** Infectious Disease Epidemiology, Robert Koch Institute, Seestraße 10, 13353 Berlin, Germany

**Keywords:** Environmental epidemiology, Infection trends, Public health, SARS-CoV-2, Time series analysis

## Abstract

This article presents a harmonized dataset combining environmental and epidemiological indicators to monitor COVID-19 activity across Germany over 131 consecutive weeks (July 2022–December 2024). The dataset integrates population-weighted SARS-CoV-2 RNA concentrations from wastewater with four independent indicators derived from clinical and participatory surveillance systems: incidence estimates from the German notification system, self-reported incidence estimates from the *GrippeWeb* participatory system, incidence estimates derived from the combination of *GrippeWeb* acute respiratory infection data with virological SARS-CoV-2 positivity rates, and incidence estimates based on primary care COVID-19 diagnoses adjusted for healthcare-seeking behavior. All case-based indicators are provided as weekly incidence estimates per 100,000 inhabitants or active participants, where applicable, and are aligned to calendar weeks starting on Monday. Wastewater values represent viral loads measured in gene copies per litre of wastewater and summarize samples collected from the previous Thursday through the current Wednesday. The dataset allows for the assessment of relationships between wastewater signals and case-based indicators of disease activity. This article focuses on dataset construction, harmonization, and structural characteristics, including temporal alignment challenges, and outlines potential analytical use cases.

Specifications Table

**Table:** 

Subject	Health Sciences, Medical Sciences & Pharmacology
Specific subject area	Wastewater-based surveillance (WBS) for SARS-CoV-2; linkage to case-based surveillance
Type of data	Raw and processed data
Data collection	Weekly data were collected from mid-2022 to December 2024. Wastewater samples were collected from wastewater treatment plants across Germany. SARS-CoV-2 viral loads were quantified by laboratories and subsequently normalized and aggregated over all treatment plants. Four indicators from case-based surveillance systems were used to reflect COVID-19 incidence in Germany: 1. The German notification system incidence. 2. Self-reported incidence from a participatory system. 3. Incidence based on a participatory system combined with positivity rates stemming from a virological sentinel system. 4. Incidence based on data from a sentinel primary care system combined with data from a participatory system.
Data source location	Institution: Robert Koch Institute
Data accessibility	Repository name: Zenodo Data identification number: DOI: 10.5281/zenodo.17959184 Direct URL to data: https://doi.org/10.5281/zenodo.17959184
Related research article	S. Abunijela, P. Pütz, T. Greiner, A.-S. Lehfeld, A. Schattschneider, U. Buchholz, J. Schumacher. Wastewater-based Surveillance as a tool for monitoring and estimating COVID-19 incidence and trends: Insights from Germany, 2022–2024. *Science of The Total Environment* 1018 (2026): 181,290.

## 1 Value of the Data

• The dataset provides weekly German time series data from five different surveillance systems for SARS-CoV-2 or COVID-19 covering 131 consecutive calendar weeks from July 2022 to December 2024. It integrates one population-weighted wastewater SARS-CoV-2 RNA indicator with four case-based indicators derived from notification, participatory, virological sentinel, and primary care surveillance systems.
• The harmonized weekly structure enables direct comparison of environmental and case-based indicators despite differences in data sources, measurement concepts, population coverage, reporting practice, and temporal reference periods.
• The dataset can be used to examine the responsiveness and interpretability of wastewater-based SARS-CoV-2 surveillance in relation to established case-based surveillance systems across different epidemiological phases, including periods of reduced testing intensity, underreporting, or shifts in healthcare-seeking behavior.
• The dataset supports analyses of temporal trends, correlations, time lags, and divergences between wastewater viral loads and COVID-19 incidence estimates. It may support researchers to develop and validate statistical models for nowcasting or forecasting COVID-19 incidence in Germany.
• The dataset is openly accessible and provides a reproducible source for researchers, public health professionals, students, and policymakers interested in time series analysis, surveillance evaluation, and teaching applications using real-world epidemiological data.

## 2 Background

Monitoring infectious disease dynamics typically relies on multiple surveillance systems that capture different aspects of disease occurrence. These systems may be influenced by factors such as testing availability, healthcare-seeking behavior, and reporting practices, which can change over time and affect the interpretation of incidence estimates. Wastewater-based surveillance (WBS) provides an additional, population-level signal by measuring pathogen concentrations independent of individual testing [1]. As such, it offers a complementary perspective to case-based indicators, particularly in situations where clinical surveillance may be incomplete or delayed.

The dataset presented in this article was developed to enable joint analysis of wastewater viral load and multiple case-based indicators of COVID-19 in Germany. It combines information from five surveillance systems into a harmonized time series, addressing differences in timing, aggregation, and measurement approaches across systems. By integrating these data sources, the dataset enables comparisons of trends, assessment of consistency between systems, and analysis of the relationship between environmental and epidemiological indicators. It is intended to support methodological work, including time series analysis, nowcasting, and evaluation of surveillance performance.

This article and the dataset it describes accompany an original research article published in Science of the Total Environment entitled “Wastewater-based Surveillance as a tool for monitoring and estimating COVID-19 incidence and trends: Insights from Germany, 2022–2024” [2].

## 3 Data Description

The dataset contains weekly observations from five surveillance systems tracking SARS-CoV-2 or COVID-19 in Germany during the 131-week observation period starting in July 2022.

The included variables are listed in Table 1 and described in more detail below.

**Table 1:** Description of variables in the present dataset.

Variable Name	Description	Data Type
Week	Calendar week starting on the indicated Monday	Date
GNS-I	German Notification System Incidence of COVID-19	Numerical
PS-SR-I	Participatory System Self-Reported	Numerical
PS-VPR-I	Participatory System combined with Virological Positivity Rate	Numerical
PC—COVID-ARI-I	Sentinel for Electronic Recording of Diagnoses of Acute Respiratory Infections, combined with Participatory System	Numerical
WW-VL	Wastewater Viral Load	Numerical

Wastewater viral load data are a nationally aggregated average consisting of data from all participating WWTPs. Four indicators derived from established surveillance systems represent COVID-19 incidence in Germany:

1. German Notification System Incidence (GNS-I): Weekly incidence of laboratory-confirmed SARS-CoV-2 infections reported through the German mandatory notification system.
  The 7-day incidence represents how many new cases were reported within the last seven days per 100,000 inhabitants [3]:
  $$\text{GNS}-I=\frac{\text{Number}\;\text{of}\;\text{confirmed}\;\text{cases}}{\text{Population}}*100,000.$$
  Example: If 100,000 cases were reported in the last seven days in a population of 80 million, then
  $$\text{GNS}-I=\frac{100,000}{80,000,000}*100,000=125.$$
2. Participatory System Self-Reported Incidence (PS-SR-I): Incidence based on self-reported symptomatic COVID-19 cases from the *GrippeWeb* participatory surveillance platform.
  Participants report weekly; thus, the weekly incidence per 100,000 inhabitants is:
  $$\text{PS}-\text{SR}-I=\frac{\text{New}\;\text{self}-\text{reported}\;\text{symptomatic}\;\text{COVID}-19\;\text{cases}}{\text{Total}\;\text{active}\;\text{participants}}*100,000.$$
  Example: If 50 out of 10,000 participants self-report SARS-CoV-2 infection, then
  $$\text{PS}-\text{SR}-I=\frac{50}{10,000}*100,000=500.$$
  Data were additionally weighted according to sex, age group and federal state.
3. Participatory System combined with Virological Positivity Rates Incidence (PS-VPR-I): Incidence estimate combining weekly reported syndromic data on ARI (acute respiratory infections) from the *GrippeWeb* participatory surveillance platform (weighted according to sex, age group and federal state) and SARS-CoV-2 positivity rates from the virological sentinel surveillance system (weighted according to age group and federal state).
  As ARI can be divided into ILI (influenza-like illness) and non-ILI and positivity rates change by syndrome, PS-VPR-I for ILI and non-ILI are calculated separately, then added up and normalized to obtain an incidence estimate per 100,000 inhabitants [4]:
  $$\begin{array}{ccc} \text{PS}-\text{VPR}-I_{\text{ILI}} & = & \text{ILI}-\text{rate}*\text{SARS}-\text{CoV}-2\;\text{PR}\;\text{among}\;\text{ILI}-\text{patients},\\ \text{PS}-\text{VPR}-I_{\text{non}-\text{ILI}} & = & \text{non}-\text{ILI}-\text{rate}*\text{SARS}-\text{CoV}-2\;\text{PR}\;\text{among}\;\text{non}-\text{ILI}-\text{patients},\\ \text{PS}-\text{VPR}-I & = & (\text{PS}-\text{VPR}-I_{\text{ILI}}+\text{PS}-\text{VPR}-I_{\text{non}-\text{ILI}})\cdot 100,000 \end{array}$$
  Example: If 4% of *GrippeWeb* participants report ILI and 10% of sentinel samples among ILI-patients test positive, and 6% of *GrippeWeb* participants report non-ILI and 20% of sentinel samples among non-ILI-patients test positive, then:
  PS−VPR−I=(0.04*0.10+0.06*0.20)*100,000=1,600.
4. Primary Care Sentinel for Electronic Recording of Diagnoses of Acute Respiratory Infections, combined with Participatory System (Primary Care–COVID-ARI-I, PC—COVID-ARI-I): Incidence derived from sentinel primary care data on COVID-19 diagnoses, adjusted for healthcare-seeking behavior data from the *GrippeWeb* participatory surveillance platform.
  Weekly population-level incidence is calculated by dividing the 7-day incidence of ICD-coded COVID-19 diagnoses per 100,000 inhabitants in primary care (weighted according to age group and federal state) by the proportion of persons with ARI consulting a physician in the last seven days:
  $$\text{PC}-\text{COVID}-\text{ARI}-I=\frac{\text{COVID}-19\;\text{incidence}\;\text{in}\;\text{primary}\;\text{care}}{\text{Proportion}\;\text{of}\;\text{ARI}\;\text{patients}\;\text{consulting}\;\text{physicians}}.$$
  Example: If, in the last seven days, 200 COVID-19 consultations occur per 100,000 and 40% of ARI cases seek medical care,
  $$\text{PC}-\text{COVID}-\text{ARI}-I=\frac{200}{0.4}=500.$$

A more detailed description of these systems, including data acquisition, can be found in the accompanying research article and the references therein [2].

**Fig. 1:**
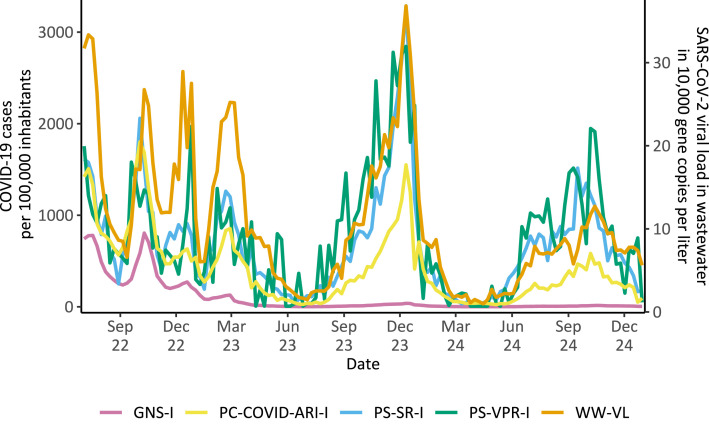
Course of the data from the five surveillance systems over time. GNS-I = German Notification System of COVID-19, PC—COVID-ARI-I = Primary Care Sentinel for Electronic Recording of Diagnoses of Acute Respiratory Infections, combined with Participatory System, PS-SR-I = Participatory System Self-Reported, PS-VPR-I = Participatory System Virological Positivity Rate, WW-VL = Wastewater Viral Load.

The course of the five indicators over time is displayed in Fig. 1.

## 4 Experimental Design, Materials and Methods

All data underlying the calculation of the indicators were collected either by the Robert Koch Institute or through surveillance systems coordinated by the Robert Koch Institute. The unique characteristic of the dataset is the retrospective integration of these five independently collected indicators into one harmonized nationwide weekly time series for Germany.

Wastewater data were generated within the German national wastewater surveillance framework. Participating wastewater treatment plants across Germany collected samples according to provided technical guidelines. Samples were analysed by participating laboratories for SARS-CoV-2 RNA using reverse transcription quantitative PCR (RT-qPCR). Viral loads were reported as gene copies per litre of wastewater. For the present dataset, measurements from individual wastewater treatment plants were normalized and aggregated into a nationwide population-weighted indicator reflecting SARS-CoV-2 RNA levels in the population.

The four indicators stem from case-based surveillance systems with different data-generating processes, including mandatory notification of laboratory-confirmed infections, weekly participant reports, virological sentinel testing, and sentinel primary care diagnoses. Only aggregated indicator values were included in the final dataset. Each case-based indicator was transformed into weekly incidence estimates using the definitions and weighting procedures of the respective surveillance systems.

To ensure harmonization across surveillance systems, all indicators were assigned to calendar weeks starting on Monday. However, the temporal reference period differs across systems. For GNS-I, the assigned calendar week corresponds to the reporting date within the notification system. For PS-SR-I and PS-VPR-I, the assigned week corresponds to the week of illness onset reported within the *GrippeWeb* participatory surveillance platform as well as the week of consultation for PC—COVID-ARI-I. For the last indicator, PC—COVID-ARI-I, the assigned week corresponds to the consultation week, while accounting for illnesses occurring during the preceding two weeks. The wastewater viral load (in gene copies/L) includes data from the previous week’s Thursday to the current Wednesday of the indicated calendar week. This alignment was chosen to retain the original reporting structure while allowing comparison across indicators with different temporal reference periods.

The final dataset was generated by merging the five harmonized indicators by calendar week. Thus, it contains aggregated nationwide weekly values and allows reproducible analyses of temporal trends, correlations, lagged associations, and agreement between wastewater-based and case-based indicators of COVID-19 activity in Germany.

## Limitations

All surveillance systems included are subject to reporting delays, which are not accounted for in this dataset. Thus, data from a given week are not necessarily consistent with the actual data that was available at that particular point in time. Likewise, methodological changes in the surveillance systems or in the derivations of the indicators after the end of 2024 are not considered in this dataset and may impair the comparison with more recent indicators derived from the same reporting systems. Differences in underlying measurement concepts (infection vs. symptoms vs. care-seeking) may further affect direct comparability across indicators.

Further limitations of the individual surveillance systems and corresponding data can be found in the accompanying research article [2].

## Ethics Statement

All authors have read and followed the ethical requirements for publication in Data in Brief. The current work does not involve individual-level data on human subjects, animal experiments, or any data collected from social media platforms.

## CRediT Author Statement

**Peter Pütz**: Conceptualization, Software, Data Curation, Writing – Original Draft, Writing – review & editing, Visualization, **Susan Abunijela**: Conceptualization, Methodology, Data acquisition, Writing – review & editing, **Udo Buchholz**: Conceptualization, Methodology, Data acquisition, Writing – review & editing, **Timo Greiner**: Funding acquisition, Project administration, Writing – review & editing, **Ann-Sophie Lehfeld**: Data acquisition, Writing – review & editing, **Alexander Schattschneider**: Data acquisition, Writing – review & editing, **Jakob Schumacher**: Conceptualization, Methodology, Data acquisition, Writing – review & editing.

## Data Availability

ZenodoWastewater-based and case-based Surveillance data for monitoring COVID-19 in Germany, 2022–2024 (Original data).
